# Analysis of the Surface of Historic Fabric from the Auschwitz-Birkenau State Museum after Treatment with Ethanol Mist Used to Eliminate Microorganisms Harmful to Human Health

**DOI:** 10.3390/ma17102323

**Published:** 2024-05-14

**Authors:** Anna Wawrzyk, Janina Poskrobko, Krystyna Guzińska, Dorota Kaźmierczak, Aleksandra Papis, Nel Jastrzębiowska, Natalia Uroda, Maria Szymankiewicz, Dagmara Zeljaś, Iga Wawrzyk-Bochenek, Sławomir Wilczyński

**Affiliations:** 1 Silesian Park of Medical Technology Kardio-Med Silesia in Zabrze, M. Curie Skłodowskiej 10C Str., 41-800 Zabrze, Poland; 2 Auschwitz-Birkenau State Museum, Więźniów Oświęcimia 20, 32-603 Oświęcim, Poland; 3 The Department of Textile Conservation, The Metropolitan Museum of Art (The Met), 1000 Fifth Avenue, New York, NY 10028, USA; janina.poskrobko@metmuseum.org; 4 Lukasiewicz Research Network-Lodz Institute of Technology, M. Sklodowskiej-Curie 19/27, 90-570 Lodz, Poland; 5 Department of Microbiology, Prof. F. Łukaszczyk Oncology Centre, 85-796 Bydgoszcz, Poland; 6 Department of Drilling and Geoengineering, Faculty of Drilling, Oil and Gas, AGH University of Krakow, Al. A. Mickiewicza 30, 30-059 Krakow, Poland; dagzel@agh.edu.pl; 7 Department of Basic Biomedical Science, Faculty of Pharmaceutical Sciences in Sosnowiec, Medical University of Silesia, Kasztanowa 3, 41-205 Sosnowiec, Poland

**Keywords:** ethanol mist, disinfection, microorganisms, FTIR, XPS

## Abstract

Aim: the aim of the work was to present the changes occurring on the model and historical cotton surface of cotton resulting from disinfection with 90% ethanol mist. Materials and methods: Samples of historical materials consisted of fabric elements from suitcases stored in A-BSM. A mist of 90% ethanol was applied for 15 s at a distance of 16 cm from the surface. The spectra of cotton samples before and after ethanol application were recorded using Fourier transform infrared spectroscopy (FTIR-ATR). Analyses of the surface layers were performed using X-ray photoelectron spectroscopy (XPS). Results: the decontamination performed did not show any significant differences in the chemical composition and surface structure of cotton before and after the use of 90% ethanol mist. Conclusions: Ethanol mist, which eliminates microorganisms from the historical surface, does not cause significant changes to the surface of historical objects.

## 1. Introduction

Preserving cultural heritage for years is very important for a country like Poland. The state of preservation of historical objects depends on the protection works, among others: disinfection that protects against biodeterioration.

KL Auschwitz was the largest concentration camp and extermination center established by Nazi Germany during World War II on the outskirts of Oświęcim in occupied Poland. Auschwitz was originally intended to serve as concentration camp and a place of slow death for Polish political prisoners and other Poles. In later years, however, it gradually became the main center of mass extermination of European Jews and the largest concentration camp for prisoners of other nationalities from almost all of Europe. KL Auschwitz is currently the most recognizable symbol of terror, genocide, and the Shoah.

A total of at least 1,300,000 people were deported to Auschwitz; 1,000,000 of them died [1]. 

**Figure 1:**
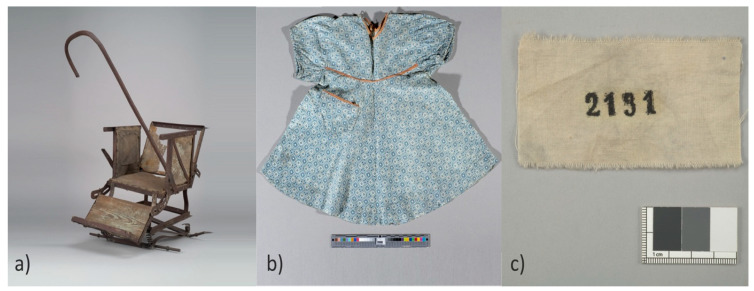
Cotton objects from the collections of the Auschwitz-Birkenau State Museum in Oświęcim. A child’s carriage with elements made of cotton fabric (**a**), and objects entirely made of cotton: a child’s dress (**b**), and a prisoner’s camp number patch (**c**).

After the Second World War, following the liberation, the Auschwitz-Birkenau State Museum in Oświęcim (A-BSM) was established at the site of the former concentration camp, focusing on preserving the remnants of the Auschwitz-Birkenau Konzentrationslager. During the museum’s formation, everything found within the area of the former camp structure was secured to the best extent possible, laying the foundation for the museum’s collection. Personal items discovered after liberation often serve as the sole testament to the presence of individuals deported to KL Auschwitz. A significant part of these objects, known as looted property, comprises items containing fabric in their composition. These include, among others: clothing, shoes, prosthetics, belts, and suitcases. The presence of fabric in individual objects varies. Objects can be entirely made of fabric, while others incorporate fabric as one of several materials used in their creation (Figure 1).

A significant part of the collection is in poor condition, primarily due to the prolonged use of items and the way they were treated after confiscation in the camp. They were often piled in large heaps in front of warehouse buildings, directly on the ground. Additional damage occurred during the searching process after returning them to their owners, leading to numerous instances of mechanical destruction.

The main conservation efforts at A-BSM regarding textile objects focus on appropriate prevention, ensuring optimal storage and exhibition conditions, including microbiological cleanliness. In order to minimize the potential spread of microorganisms, suitable decontamination techniques are sought. A-BSM is testing various disinfection techniques for historical objects using different methods and biocidal substances. The primary goal of these investigations is to eliminate microorganisms harmful to human health and those acting destructively on surfaces isolated from historical objects stored in the Collection Department. This is crucial for the preservation of cultural heritage, as confirmed by numerous studies on the biodegradation of cultural heritage [2,3,4,5].

The species composition of microorganisms varies depending on the material they inhabit. The microbiome composition is largely determined by the type of substance covering the historical material. Therefore, in the field of museology, constant efforts are made to find new disinfection methods. In each case, the biocidal effectiveness of a method depends on the properties of the disinfected surface material. Often, disinfection methods are adapted from the medical field, where they were applied to abiotic surfaces [6].

Microbiological studies conducted in museums worldwide and at A-BSM have revealed the presence of various types of bacteria on the surfaces of textiles. One frequently encountered type is the spore-forming *Bacillus* sp. with cellulolytic potential [1,7,8].

These rods can be harmful to historical objects, and also exhibit pathogenic potential. *B. cereus* is a common cause of acute food poisoning and post-traumatic eye infections, while *B. subtilis* has been isolated from individuals with bacteremia, endocarditis, pneumonia, and sepsis [9,10].

On the surface of historical textiles at A-BSM, 12 species of fungi were detected, including *Aspergillus flavus* and *Aspergillus niger* [11].

*A. flavus* is the main etiological agent of allergic bronchopulmonary aspergillosis and lung infections. It has also been linked to infections of the ear canal, pulmonary aspergillosis, and other localized aspergillosis [12,13]. 

The elimination of *A. flavus* from the museum environment is essential because, under favorable conditions, this fungus produces aflatoxins in large quantities, which are hepatotoxic and carcinogenic [14]. 

*A. niger*, when detected on objects, is responsible for infections of the middle and external ear, as well as nail fungus. It causes invasive pulmonary aspergillosis, and certain strains can lead to subcutaneous infections [15,16]. 

Fungi isolated from the museum environment can, under favorable conditions, produce toxic aflatoxins and ochratoxins, like other mycotoxins, posing health risks [17].

*A. niger* is highly pervasive and invasive, posing a threat not only to humans but also to historical objects. Even one colony of this fungus detected on an object at A-BSM qualifies the object for comprehensive disinfection.

Given that potentially harmful microorganisms are isolated in museum environments, A-BSM tested the biocidal effectiveness of vaporized hydrogen peroxide (VHP), ethylene oxide (EtO), and diode laser. Each of these techniques is dedicated to different types of objects. VHP and EtO are used for comprehensive disinfection of large object surfaces through fogging [11].

A diode laser was tested for the elimination of microorganisms on the surfaces of historical textiles appearing as very small spots [18].

Research conducted currently at A-BSM focuses on adapting the ethanol mist disinfection method to surfaces of approximately 1 m or slightly larger, which is the subject of this study. To eliminate fungi and bacteria with potential pathogenic and cellulolytic capabilities, 90% ethanol mist has been applied. Wawrzyk et al., presented the results of microbiological studies, confirming the biocidal effectiveness of the method. The reduction of most microorganisms was over 99% [19].

To confirm that ethanol mist does not have a harmful effect on the fibers of historical fabric, the authors conducted scanning electron microscopy (SEM) tests, which did not show any significant, visible changes on the surface after the use of ethanol mist [19].

In the case of employing innovative techniques or using a biocidal agent different from the traditional one, the composition and structure of the disinfected material should not undergo adverse changes. The authors also presented results from Scanning Electron Microscopy (SEM), which did not reveal significant, visible changes on the surface after the application of ethanol mist [19].

For a more detailed analysis of the impact of ethanol mist on the chemical properties of historical materials’ surfaces, Fourier-Transform Infrared Spectroscopy (FTIR) analysis was conducted before and after the decontamination process. FTIR is successfully used for analyzing textile surfaces, as confirmed by researchers [20].

It has also been applied to analyze historical paper [21].

In A-BSM, this research technique has been previously used for the analysis of textiles, leather, and materials based on cellulose nitrate [18].

Researchers also recommend this technique for archaeological textile studies because it is non-invasive and non-destructive [22].

To further assess the potential impact of ethanol mist on the near-surface layer at PMA-B, X-ray photoelectron spectroscopy (XPS) studies were conducted, allowing for the capture of changes up to a depth of 2–3 nm. XPS is successfully utilized for studying both biotic and abiotic surfaces [23].

Topalovic applied XPS to determine chemical changes occurring on the surface of cotton due to bleaching, explaining the correlation between the increase in the capillary constant and the removal of non-cellulosic impurities characterized by the C1 component in the C1s XPS spectrum [24].

XPS has also been used for the analysis and conservation studies of artistic emulsion acrylic paints [25].

In A-BSM and other museums, it is extremely important to eliminate from the environment microorganisms that are potentially harmful to human health and those that may contribute to the biodegradation of objects.

Therefore, the aim of the work is to analyze, using highly specialized methods, the potential impact of 90% ethanol mist on the chemical changes that may occur on the surfaces of highly degraded historic materials.

## 2. Materials and Methods

### 2.1. Research Objects

The surfaces of both the model and historical materials underwent Fourier-Transform Infrared Spectroscopy (FTIR Thermo Fisher Scientific, 168 Third Avenue Waltham, MA, USA, 02451) and X-ray photoelectron spectroscopy (XPS, 100 Red Schoolhouse Road, Bldg. A-8 Chestnut Ridge, NY 10977, USA) analyses. The model material used was cotton (SDC Enterprises Limited, Thongsbridge, UK) with a grammage of 100 g/m^2^. Before the decontamination effectiveness test, the samples were sterilized (121 °C, 20 min).

The historical material sample was cut from the inner side of a suitcase lid from the collections of A-BSM, dating back to the first half of the 20th century. Fiber analysis revealed that it was dusty cotton covered with secondary conservation agents.

The dimensions of the model fabric samples were adjusted to the dimensions of the historic fabric and selected so that the total surface was 100 cm ^2^. Two cotton samples with dimensions of 50 × 100 mm and two cotton samples with dimensions of 40 × 12.5 mm on which ethanol was applied were used as controls. The test was performed in two repetitions.

### 2.2. Application of 90% Ethanol in Mist Form on Cotton

Both short-term and long-term effects of 90% ethanol (Chempur, Piekary Śląskie, Poland) were investigated. For this purpose, ethanol mist was applied to the samples using Paasche VL 0819 and VE 0707 airbrushes at a pressure of 0.2 MPa and a PA HEAD VLH-5 nozzle (with a diameter of 1.05 mm). The application was carried out in a chamber with laminar air flow of microbiological safety class II, with a double HEPA filtration system ensuring the flow of sterile air. During application, the sample was placed vertically and secured with a metal handle. As part of the optimization of the method, the pressure parameters, nozzle and application time were adjusted to ensure the minimum moisture of the samples. After applying ethanol, the sample was weighed to determine the mass of ethanol applied.

During the assessment of the short-term effect of ethanol, the mass of the applied solution depended on the alcohol concentration and ranged from 0.2 to 1.0 g per 100 cm^2^. The application time was set at 4–16 s/100 cm^2^. Subsequently, the cotton was dried in a sterile chamber. To examine the long-term effect of ethanol, after the short-term treatment, the contact time of microorganisms with ethanol was extended. Following the application, the cotton was wrapped in foil, and after 22 h, it was also dried in a sterile chamber.

### 2.3. Chemical Analysis of the Surfaces

#### 2.3.1. FTIR Analysis of the Chemical Composition of Cotton Surface before and after Decontamination with Ethanol

To investigate whether ethanol induced adverse changes on the surface of the disinfected fabric, Fourier-Transform Infrared Spectroscopy (FTIR) analysis was employed. The FTIR analysis was conducted using a Nicolet 8700 FTIR spectrometer (Thermo Scientific, Waltham, MA, USA) with an Attenuated Total Reflection (ATR) diamond crystal and a liquid nitrogen-cooled Mercury-Cadmium-Telluride (MCT-A) detector. Spectra were collected before and after the application of ethanol mist from a layer with a thickness of 2–3 μm in the range of 4000–650 cm^−1^ and with a resolution of 4 cm^−1^. ATR spectra underwent ATR correction, scaled normalization, and baseline correction. The obtained spectra were equivalent to transmittance spectra. The software OMNIC 3.2 (Thermo Scientific, USA) was utilized for the analysis.

#### 2.3.2. XPS Analysis of the Chemical Composition of Cotton Surface before and after Decontamination with Ethanol

For the analysis of the near-surface layer of materials before and after decontamination, X-ray photoelectron spectroscopy (XPS) was employed. The analysis utilized a multi-chamber Ultra High Vaccum (UHV) analytical system (Prevac, Poland). Photoelectrons were excited by X-rays with Al Kα characteristic line at an energy of 1486.7 eV, generated by a VG Scienta SAX 100 lamp with an aluminum anode along with a VG Scienta XM 780 monochromator. The X-ray lamp operated at U = 12 kV and Ie = 30 mA. Photoelectrons were recorded using a hemispherical analyzer Scienta R4000. The pressure in the analysis chamber during measurements was below 1.0 × 10^−8^ mbar. The fundamental parameters for the survey spectrum were as follows: sweeping mode, pass energy: 200 eV, measured range of photoelectron binding energy: 0–1350 eV, step size: 0.5 eV, and dwell time in a single step: 200 ms. To compensate for the electric charge formed during measurement, samples were bombarded with a low-energy electron beam. The recorded spectra were processed using Casa XPS Version 2.3.16 PR16 software. All spectra were calibrated by establishing the position of the C1s carbon line at an energy of 284.5 eV.

#### 2.3.3. Quality Assurance in Research

Quantitative microbiological methods were validated by repetition test 20 times. The accuracy values were <0.25 log under repeatability conditions and <0.33 log under intralaboratory reproducibility conditions. The workload under repeatability conditions was also determined for all physicochemical elements of quantitative methods, and amounted to 1% for FTIR and 7% for XPS. Moreover, to ensure quality in ethanol biocidal effectiveness testing, each combination of microbial strain and ethanol mist parameter variant was performed using three samples, and each sample was tested twice. FTIR and XPS spectra were collected for one area of each material at a number of replicates appropriate for each method.

## 3. Results

### 3.1. Fourier-Transform Infrared Spectroscopy (FTIR) Analysis of the Surface of Disinfected Model and Historical Cotton

The first stage of research, focusing on the analysis of the impact of 90% ethanol mist decontamination on the historic cotton surface, involved conducting measurements using the FTIR-ATR technique. The application of this method aimed to determine the influence of the applied biocidal solution on the chemical structure of the decontaminated surface. Three samples of model cotton and three samples of historical cotton were used for the study. The model cotton, with a known and precisely defined chemical structure, served as a reference material in relation to the historical cotton, which was the target material for the discussed decontamination method. Samples of historical materials are available in small quantities because they are cultural heritage, which is the greatest limitation in research conducted on historical objects.

Sample I of the model cotton and historical cotton served as control samples not subjected to the decontamination process. Sample II of the model cotton and historical cotton was prepared by applying 90% ethanol mist to the material surface, and measurements were taken immediately after the biocidal agent dried. The last samples, i.e., model cotton III and historical cotton III, were prepared similarly to samples II, but the measurements were taken after 22 h of decontamination. During this time, these samples were stored in a closed container. 

#### 3.1.1. Fourier-Transform Infrared Spectroscopy (FTIR) Analysis of the Surface of Disinfected Model Cotton

The model material is used for preliminary research so as not to destroy the monuments. It has properties similar to disinfected historical materials, but does not have a layer of dust or impregnation on the surface.

**Figure 2:**
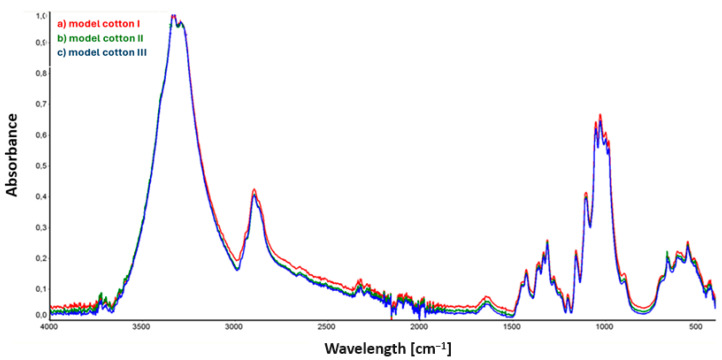
ATR collective spectra of samples: (a) model cotton I (sample not subjected to decontamination); (b) model cotton II (sample subjected to 90% ethanol mist decontamination); (c) model cotton III (sample subjected to 90% ethanol mist decontamination and stored in foil for an additional 22 h).

As a result of conducting FTIR measurements using the ATR attachment, spectra were obtained for the model material samples, which are presented in Figure 2 below. Figure 2 below shows the collective spectrum for three model cotton samples.

#### 3.1.2. Fourier-Transform Infrared Spectroscopy (FTIR) Analysis of the Surface of Disinfected Historical Cotton

**Figure 3:**
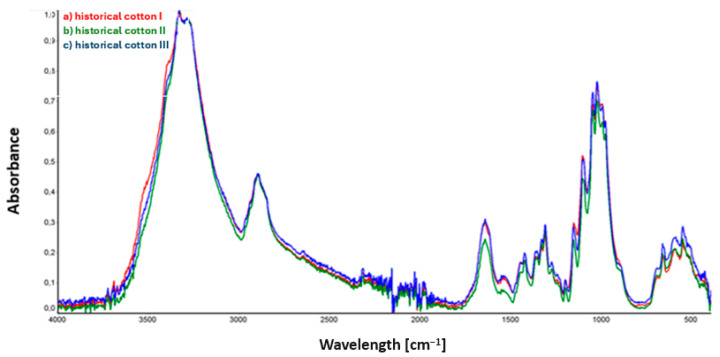
ATR collective spectra of samples: (a) historical cotton I (sample not subjected to decontamination); (b) historical cotton II (sample subjected to 90% ethanol mist decontamination); (c) historical cotton III (sample subjected to 90% ethanol mist decontamination and stored in foil for an additional 22 h).

The spectra obtained from the analysis of historical material are presented in Figure 3.

Confirmation of the relationship between the peak intensities for the three historical cotton samples shown in the spectra (Figure 3) is presented in the composite spectrum containing graphs for the three historical cotton samples mentioned.

Analysis of the spectra from samples of model cotton, presented in Figure 2, did not reveal noticeable and significant differences in the intensity and position of spectral bands in the IR spectrum.

In the case of historical cotton samples (Figure 3), the only visible change in the spectrum concerns the intensity of bands in the range of 1650–1730 cm^−1^, corresponding to stretching vibrations of C=O groups. Reduced intensity of the band around 1650 cm^−1^ is observed for historical cotton sample II, and may result from incomplete evaporation of the applied 90% ethanol solution on the surface of the examined material. The peaks of the other two samples, historical cotton I and historical cotton III, located at the same wavenumber value, overlap with each other.

### 3.2. X-ray Photoelectron Spectroscopy (XPS) Analysis of the Surface of Decontaminated Model and Historical Cotton

#### 3.2.1. X-ray Photoelectron Spectroscopy (XPS) Analysis of the Surface of Decontaminated Model Cotton

**Figure 4:**
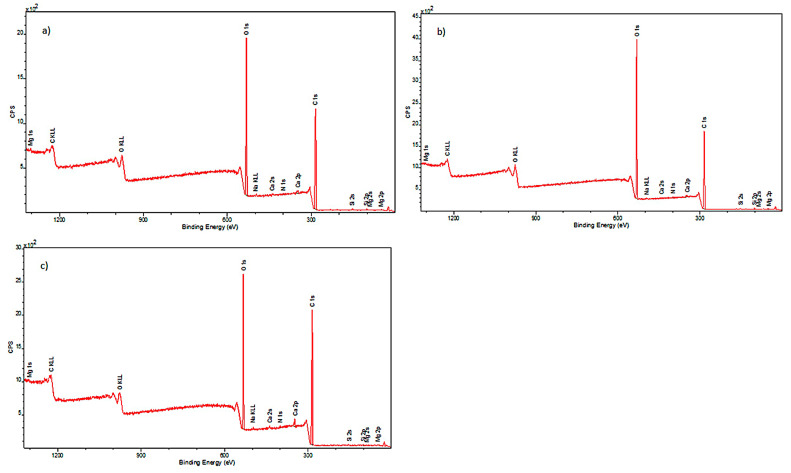
XPS spectra obtained for samples: (**a**) model cotton I (sample not subjected to decontamination); (**b**) model cotton II (sample subjected to decontamination with 90% ethanol mist); (**c**) model cotton III (sample subjected to decontamination with 90% ethanol mist and additionally stored in foil for 22 h).

To fully illustrate the impact of decontamination with a 90% ethanol mist on cotton surfaces, a complementary study was conducted using XPS. It allowed the determination of the elemental composition of the external layers of the investigated model samples. The obtained spectra are presented in Figure 4.

#### 3.2.2. X-ray Photoelectron Spectroscopy (XPS) Analysis of the Surface of Decontaminated Historical Cotton

**Figure 5:**
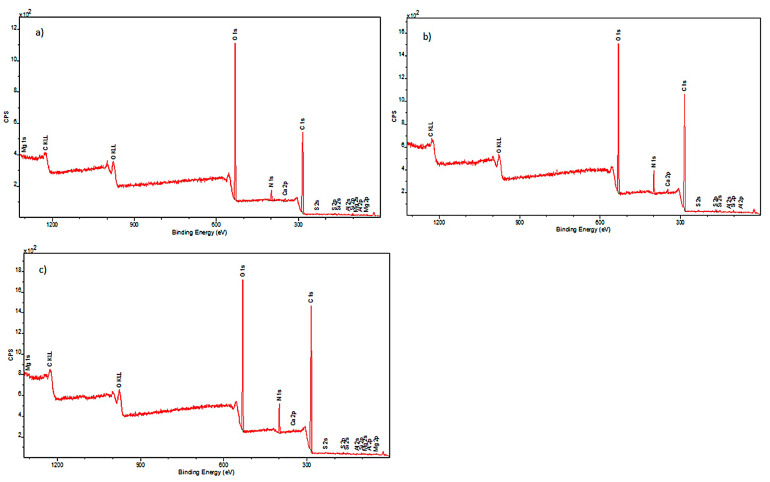
XPS spectra for samples: (**a**) historical cotton I (not subjected to disinfection); (**b**) historical cotton II (sample subjected to disinfection with 90% ethanol in the form of mist); (**c**) historical cotton III (sample subjected to disinfection with 90% ethanol in the form of mist and additionally stored in foil for 22 h).

Elemental composition of historical cotton was also determined. The obtained spectra are presented in Figure 5.

Analysis of the spectral lines visible in the spectra allowed for the detection of elements present in the examined samples. The outer surface of the model cotton consisted of the following elements: carbon, nitrogen, oxygen, magnesium, silicon, and calcium. The historical cotton samples additionally contained aluminum and sulfur on their surface.

**Figure 6:**
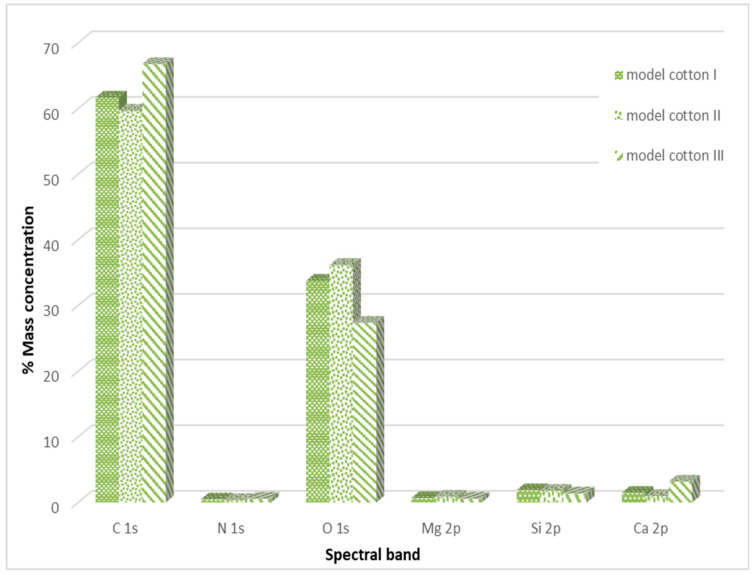
Percentage distribution of individual elements in the outer layer of the examined model cotton samples.

Detailed information regarding the percentage distribution of individual elements on the model cotton, obtained in the course of the conducted study, is presented in Figure 6.

**Figure 7:**
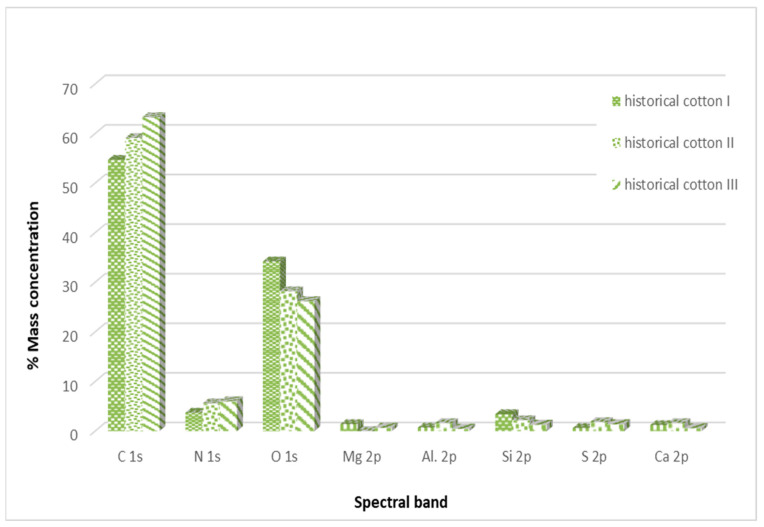
Percentage distribution of individual elements in the outer layer of the examined historical cotton samples.

Figure 7 illustrates the percentage distribution of individual elements on historical cotton obtained during the XPS study.

It was observed that, in the case of model cotton samples, the vast majority of the elemental composition, expressed in mass percentages, consists of carbon and oxygen. This is due to the structure of cotton, which is composed of over 90% cellulose. XPS studies conducted in a narrow binding energy range showed the presence of the following chemical bonds characteristic of carbon: C-C, C-O-C, C=O, O-C=O. Calcium present in the samples occurs in the form of carbonates, while silicon is present in the form of silicon dioxide. No significant differences were observed between the model cotton samples not subjected to decontamination and those treated with 90% ethanol in the form of mist.

Historical cotton samples also show the highest content of carbon and oxygen in their composition. Additionally, an increase in nitrogen content was observed compared to model cotton samples, which likely originates from the chemical compounds used for dyeing materials, such as azo dyes, nitroso dyes, or nitric dyes [26].

In the case of historical samples, aluminum and sulfur were additionally detected, which most likely constitute a secondary layer of the examined historical surface. The analysis of historical cotton material samples also did not reveal significant changes in the elemental composition after decontamination with a 90% ethanol mist.

The increase in carbon content in both types of material and oxygen content in the case of model material is most likely due to the incomplete evaporation of ethanol applied to the examined surfaces. Ethanol has carbon and oxygen atoms in its structure, which can be detected during surface studies.

The results were obtained after applying ethanol mist from the same distance, but the limitation of the method will be its repeatability in real conditions, when the airbrush is used by conservators freely. You cannot then maintain a constant distance.

## 4. Discussion

In A-BSM and other museums, microorganisms harmful to human health and those that can adversely affect the surfaces of historical objects are isolated. In museology, in addition to the biocidal aspect, minimizing the impact of the applied decontamination on the structure and morphology of disinfected surfaces is crucial. Various decontamination methods have been tested in museums, including gamma radiation, X-ray radiation, low-temperature plasma, volatile compounds, and essential oils, as well as silver nanoparticles [27,28,29,30,31,32].

Researchers have achieved different degrees of microbial reduction. In A-BSM, the biocidal efficacy of vaporized hydrogen peroxide (VHP), ethylene oxide (EtO), diode laser, and the focus of this study, ethanol in the form of mist, was tested [11].

VHP, previously used in medical spaces, resulted in a reduction (R) of the majority of tested microbial strains by a minimum of R = 3 log and all tested mixed cultures above R = 98% when applied at a concentration of 300 ppm for 20 min on porous textile material [6].

Historical cardboard subjected to disinfection using VHP (300 ppm, 20 min) showed a reduction in the number of isolated microorganisms by 1.6–7.0 log on new cardboard, corresponding to a reduction of 97.26–100.00% [33].

A-BSM successfully achieves disinfection with a diode laser, where in the medical field, a reduction of bacteria and fungi from 60% to 100% was achieved on various materials [34,35].

On 25-year-old corroded metal, the reduction of microorganisms using a diode laser was 88.85–100% [36].

In A-BSM, historical collagen material achieved a reduction of 78–92%, and on cellulose, it achieved 90–100% reduction [18].

Among the tested methods in museology, fogging or vapor methods were included. Disinfection with Cinnamomum zeylanicum essential oil-based alcohol mist demonstrated a reduction effect of 5–7 logs on cotton and linen textiles [37].

Using a benzalkonium chloride solution at a concentration of 0.01 mg/m^3^ in vapor form showed no reduction in bacteria on wool and cotton [38].

Researchers have also explored the impact of ethanol on artifacts, primarily in liquid form. No harmful effects of 70% ethanol were observed on the tested paper, either in the short or long term [39].

Karbowska tested the biocidal effectiveness of ethanol vapors applied for 18 h on old paper, and showed an R > 4.00 log reduction of the tested fungi. In the case of *Cladosporium cladosporioides*, only a 3 h exposure to ethanol vapor was sufficient. Other fungi, including *Penicillium spinulosum*, and the most resistant strains of *Trichoderma viride* and *Chaetomidium subfimeti*, were completely eliminated after 18 h [40].

In A-BSM, using the 90% ethanol mist decontamination technique, a reduction level was achieved at concentrations of 80% and 90%, ranging from 93.27% to 99.91% for fungi and from 94.96% to 100% for bacteria, with 74.24% for *B. subtillis* [19].

Comparing the results of the biocidal effectiveness of ethanol mist to previously tested decontamination techniques in A-BSM, such as diode laser and VHP, it can be observed that the antimicrobial effect is slightly lower. Disinfection with a diode laser with an exposure power of 0.3W in continuous CW mode for 2 min in two repetitions, a 90.20–100% reduction in the number of microorganisms was achieved. However, the use of vaporized hydrogen peroxide at a dose of 300 ppm for 20 min reduced the number of microorganisms by 70–100%. The use of various decontamination techniques gives similar results in the case of fungi, but spore-forming bacterial species, especially those of the Bacillus genus, are the most difficult to combat. The tested ethanol in the form of mist also has the lowest effectiveness against these microorganisms.

However, the conducted studies are promising because, under the applied parameters of a distance of 16 cm from the object, misting for 15 s, at 2 bar pressure, and 90% concentration of applied ethanol, no changes in the morphology and chemical structure of the surface were observed.

A crucial aspect when implementing new decontamination methods on historical artifacts is the absence of an impact on surface properties and no change in the color of the treated objects. To assess color changes, the CIELab scale is used. A-BSM cannot use any decontamination technique that changes the color of the object’s surface. Both the spectrophotometric and visual methods confirmed that the tested ethanol in the form of mist did not change the color of the disinfected surfaces of the historical fabric. Researchers confirmed using this method that a bath in absolute ethanol (99.80%) for 24 h did not alter the color of white and yellow silks, and red silk velvet. After disinfection with ethanol mist, there were no changes significant for the condition of the objects. Conservators did not observe any changes with the naked eye.

To evaluate the impact of decontamination techniques on surface morphology and fiber changes, SEM electron microscopy is employed [41].

In previous studies in A-BSM, SEM was utilized, demonstrating no changes in fiber morphology after the application of ethanol mist on historical fabric [19].

FTIR is a commonly used technique in various research fields, including museum studies. Its utility has been confirmed in the examination of cotton and other fabrics, particularly in determining changes on surfaces caused by decontamination techniques. The method has been applied to investigate the impact of ethanol and isopropanol solutions on silk artifacts. FTIR results show that even after immersing silk in these solutions for 180 min, no significant chemical or physical changes are observed in the silk fibers [42].

Analysis of FTIR results indicates that VHP decontamination of cotton fabric practically does not induce changes in the structure of cotton cellulose, thus not affecting the material’s susceptibility to biodeterioration [11].

Results from FTIR analysis of cotton fabrics after decontamination with Cinnamomum essential oil vapors and low-temperature plasma show that these processes cause only minimal changes in the molecular structure of cellulose [30,37].

FTIR has also been used to study surface changes resulting from the cleaning of cotton materials. Analyses of obtained spectra allowed the for estimation of, among other things, the amount of waxes remaining on the examined fabric layer after the cleaning process [43].

For cotton fibers, the region between 1750 and 1600 cm^−1^ is most suitable for assessing cellulose degradation through oxidation, as confirmed in the current study [44].

Kavkler et al. utilized FTIR to determine the degree of biodegradation in historical textiles based on proteinaceous components stored in museums in Slovenia. More intense biodegradation processes caused by microorganisms and other degrading factors were observed in the inner part of the fibers compared to their superficial part [45].

XPS spectroscopy enabled the analysis of the surfaces of bleached cotton fibers. This study compared and identified surface chemical changes in a sample of used fabric and a model fabric previously cleaned of easily removable contaminants [24].

XPS has also been successfully used to assess the impact of VHP on cotton fabric in the medical field [6].

In the conservation of cultural heritage, including at A-BSM, XPS has been repeatedly employed to analyze the surfaces of objects. This technique provides information about changes occurring in the near-surface layer, typically 2–3 nm deep. This is crucial, as it allows for the assessment of changes not only in the base material, but also in the layers of conservation preparations covering the objects.

In the current study, FTIR measurements did not register any impact of decontamination with a 90% ethanol mist on changes in the structure of the decontaminated cotton samples, both model and historical. It is highly probable that the ethanol solution used in the study evaporated completely or to a significant extent from the surfaces of the tested materials. Cotton primarily consists of cellulose, which does not react with ethanol.

Research conducted using XPS, complementary to the FTIR measurements, confirms that the decontamination of cotton samples with a 90% ethanol mist has no impact on the chemical structure of the outer layer of cotton. Any potential changes in the percentage content of individual elements likely result from the presence of ethanol on the surface of the samples, which did not completely evaporate.

The use of these techniques in analyzing the chemical composition of cotton surfaces suggests that they are valuable methods for the research described in this article, focusing on the impact of decontaminating cotton materials with a 90% ethanol mist. The obtained results did not show any drawbacks to the application of this form of decontamination.

The tests performed showed that ethanol effectively eliminates most of the microorganisms inhabiting historical fabrics, but worse results are achieved if the sample contains spore-forming bacteria of the Bacillus genus. The next stage of research will be related to the inclusion of antibiotics, which may improve effectiveness.

## 5. Conclusions

The decontamination method using 90% ethanol mist is biocidal, and does not negatively affect the surface structure of both model and historical cotton. Therefore, it can be used for decontamination of cotton elements in historic buildings in the A-BSM area. In further research, the method should be tested on other textile materials, for example linen, viscose, silk, and wool.

The next stage will be testing of ethanol in the form of mist with the addition of antibiotics, which may prove to be more effective against bacteria of the Bacillus genus.

Ethanol fog is safe for people who disinfect moving objects because it can be carried out in a fume hood or in a safe work chamber. A-BSM is conducting research on employee safety that will allow for the disinfection of large wall surfaces in stationary facilities. The research simultaneously focuses on the safety of facilities and the protection of people performing conservation work.

Ethanol mist is an easy-to-use, cheap and, importantly, non-destructive disinfection method that can be used on cotton objects.

## Data Availability

Data are contained within the article.
